# Pennsylvania Hospital

**Published:** 1838-07-04

**Authors:** Henry H. Smith

**Affiliations:** Resident Surgeon


### CLINICAL REPORTS.

### PENNSYLVANIA HOSPITAL.

[Reported by HENRY H. SMITH, M.D., Resident Surgeon.]

REPORT OF THREE CASES OF COMPOUND FRACTURE OF THE SKULL.

1. *Case of compound fracture of the skull, bone depressed, trephined by* DR. NORRIS.

Joseph W——, æt. 10 years, was struck on the head by a large block of wood, which fell from a three story dwelling, on the 10th August, 1837. On his entrance, three hours afterwards, he had a wound of three and a half inches in the scalp, bone bare of periosteum for two inches, fractured the length of the cut, and depressed about an eighth of an inch. Patient insensible; pulse strong, corded and slow; pupils dilated; extremities cold. He was trephined immediately by Dr. Norris. On raising the bone consciousness instantly returned. The middle artery of the dura mater was cut by the trephine, but the hæmorrhage was checked by a coagula which soon formed. The scalp was drawn partially together by adhesive strips, and the wound poulticed—ordered perfect rest in a dark room, and lowest diet. Evening.—Pulse increased, skin hot and dry—lies quiet, and complains little; ordered V. S. ad f℥vi, and mist. neut. f℥vi. ant. tart. gr. ss. a table-spoonful every 2 hours.

*August 30th*—Rested tolerably; skin more moist; continue treatment.

*August 31st*—Restless during night; considerable fever; pulse increased in force and frequency; tongue dry; ordered V. S. f℥x—continue mixture, and give calomel gr. ij. every 4 hours.

*September 1st*—Fever slight, rested better, wound dry; ordered large poultice to the whole scalp, adhesive straps removed, treatment continued.

*September 4th*—Wound looks well—suppurating kindly; little pain in head; pulse slow and natural; bowels opened very freely;—ordered cretacious mixture ℥ss. every two hours;—lowest diet, and large poultice.

*September 6th*—Diarrhœa checked; tongue clean; skin natural; treatment continued.

*September 7th*—Wound granulating from bottom; suppurating very freely;—no fever;—says he feels perfectly easy, and answers questions readily;—one end of the wound brought together by straps and poultice to wound alone.

*September 10th*—Granulations even with surface; end of wound united;—ordered vegetable diet with soup.

*September 14th*—Granulations too high; appearance of fungus cerebri; general health improving; ordered compresses of lint soaked in lime water to be applied on each side of the wound, and continue treatment.

*September 15th*—Granulations reduced a little, patient sitting up in bed, and quite merry; poultice removed; compresses continued over wound.

*September 18th*—Bone for an inch to come away; granulations healthy and even; ordered simple dressings with adhesive straps, and allowed to leave his bed.

*September 29th*—Wound healed, except in the centre; bone not yet loose; same dressings; patient goes about; feels no inconvenience from the wound.

*October 6th*—Wound closing slowly; several small pieces of bone have come away.

*October 16th*—Since last date several other pieces; portions of the outer table of the skull have been removed; wound healing; health good.

*November 5th*—Discharged cured; wound healed; parts firm and resisting, and intellect good.

2. *Case of gun-shot wound of the skull; ball remaining in the brain; discharge of medullary matter; death three days afterwards.*

John D——, æt. 16 years, on the 22d October, 1837, snapped a pocket pistol of large size, which hung fire; whilst looking in the muzzle, it went off, and the ball entered the head a little above the left frontal protuberance; he was brought at once to the Hospital. At 6 P. M., about one hour after the accident, a small wound was in the forehead; no hæmorrhage; probe passes upwards and slightly backwards for four inches directly into the substance of the brain; pulse slow and irregular; breathing slow, ten in the minute, slightly stertorous; eyes inflamed; powder in conjunctiva and skin of face; perfectly sensible, and speaks readily. Immediately after admission, vomited a large quantity of dried cherries;—vomiting caused slight hæmorrhage from wound. Ordered V.S. f℥xii. mist. nent. f℥vi. f℥ss. every two hours; injection of salt and molasses and water to bowels, and poultice to wound.

*October 23d*—Head cool; complains of no pain; rested badly; slight discharge of medullary matter from wound; pulse slow, 70 in the minute, full and irregular. Ordered V. S. ad f℥xxvi.—lowest diet—nothing but bread and tea; mist. neut. f℥vi; ant. tart. gr. ss.; table-spoonful every 2 hours, and continue poultice to wound.

*October 24th*—Great jactitation; violent spasms; constant flexion of fingers and hand of right side; no power over right leg; unable to answer questions; becoming comatose; wound discharging thin serum; ordered 6 wet cups to back of neck, and a powder of calomel gr. x. ext. colocynth compound and jalap $\overline{aa}$ gr. v.; iced water to drink, and cold cloths to the head.

*October 25th*—Pulse 120, fluttering and irregular; bowels not opened; respiration stertorous and slow; spasms slight; coma increasing; ordered injections to bowels; death at 1 P. M.

*Autopsy at 4 P. M.*—Litle effusion under scalp; skull thin; no fracture around the point where the ball entered; membranes congested; considerable effusion of blood under dura mater near f℥vj. of bloody serum found in cranium; arachnoid inflamed, and slightly thickened; long sinus, extending diagonally from the front lobe of the left hemisphere of the cerebrum to the posterior lobe, through the lateral sinus, was made by the ball, the whole course of which was lined with pus; a piece of bone near the size of the ball had been driven from the os frontis into the ventricle; ventricle contained a large quantity of serum similar to that discharged from the wound; the ball was found on the posterior edge of the corpus callosum; the right hemisphere was uninjured, as was the cerebellum.

3. *Case of compound fracture of the skull, caused by an injury in a saw-mill; death in forty-eight hours; pus on the substance of the brain.*

E. H——, æt. 39 years, whilst engaged on the morning of the 13th June, 1838, attending a steam saw-mill, fell, head foremost, against a large circular saw, which was revolving at the rate of 240 revolutions in the minute. A fur cap on his head was torn to tatters, the scalp terribly lacerated, and turned back, leaving the skull bare for a space of four inches long, by three wide; the skull was fractured from the lower part of the os frontis to the vertex, in a line a little oblique to the sagittal suture. The saw had penetrated the dura mater, a process of which was hanging out, and entered deeply into the substance of the brain. The hæmorrhage had been profuse previous to entrance, and the head had been temporarily dressed by Dr. West. On his entrance, about one hour after the accident, the head was in the state mentioned. A branch of the temporal and occipital arteries was tied, the wound thoroughly cleansed, and the scalp brought together by adhesive straps; a compress of dry lint was placed over the wound, and a light bandage applied to the head. The patient remained perfectly sensible, and able to set up in bed; pulse good, and but little accelerated—complained little of pain; ordered perfect rest in a dark room, and low diet.

*Night.* Pulse a little quicker; slight cephalalgia; skin and tongue rather dry; ordered iced lemonade, and mist. neut. f℥vi., ant. tart. gr. j, f℥ss. every two hours.

*June 14th.* Has passed a restless night; tongue dry; pulse slow; tendency to delirium; answers questions slowly; passed water twice since accident; bowels not opened; ordered calomel and jalap āā gr. x; ice to the head, and continue mixture.

*Noon.* Rapidly becoming comatose; catheter drew off over a pint of water.

*Night.* Coma perfect; floccitation; extreme jactitation; urine and fæces evacuated involuntarily; powder has not operated; ordered injection to bowels, which came away immediately; treatment continued.

*June 15th.* Death at 5 A. M.

*Autopsy at half-past* 11, *A. M.*—Present Drs. Norris, West, Pepper, Wallace, and Meigs. Wound offensive, and decomposing rapidly; slight discharge from wound; skull bare for four inches, and fractured from orbitar process of os frontis to the upper part of the occipital bone; skull notched in several places, and small portions of the outer table removed by the saw. On removing the skull-cap, it was found to have been cut through for the length of three and a half inches, and fractured at each end of this space for the distance mentioned; no blood effused on dura mater; cerebral matter protruding; wound in dura mater the length of that in skull; arachnoid much inflamed and thickened; several drachms of pure pus found on the brain and membranes; vessels injected; brain very soft around the wound; wound in the anterior lobe of left hemisphere of cerebrum, two and a half inches long, and near two inches deep; brain covered with pus, and much softened on that side, though only forty-eight hours had elapsed since the injury. The remainder of the brain was sound; a few hydatids were found around the plexus choroides; otherwise natural.

---

*List of Accidents, admitted into the Pennsylvania Hospital, from June* 13*th to June* 27*th*, 1838.

One case of fracture of the upper third of the femur, caused by being caught between a steam-boat and the wharf; dressed with Dessault's splints, as modified by Dr. Physick. One case of dislocation of the femur upwards and backwards on the dorsum illii, caused by a fall from a height of ten feet, on the knee and thigh; reduced within three hours after admission, by means of the pullies, extension being continued for forty-three minutes; the bandage confining the roller to the thigh, having slipped several times, a clove hitch was formed of a sheet, which kept its place perfectly. One case of laceration of the ends of the second and third fingers, caused by being caught between two cog-wheels; dressed with poultice. One case of incised wound of the throat, caused by an attempt to commit suicide; the larynx and blood vessels were not injured; the parts were united by two sutures, and afterwards dressed with adhesive plaster; discharged cured in eleven days. One case of fracture of both bones of the leg, caused by the falling of a bank of earth; dressed with a fracture box. One case of incised wound of the back of the hand, caused by being stabbed; dressed with adhesive strips, and a splint; nearly well. One case of lacerated wound of the thigh, caused by a stone, upwards of a ton in weight, falling on it, at a quarry; a piece of the integuments was torn from the groin back to the buttock, fascia uninjured; it was sewed on with the continued suture previous to admission. A portion of the stitches were cut out to allow of the escape of matter, and the parts poulticed; the man has since recovered from an attack of mania a potu, the flap has sloughed off, and the parts are now suppurating; ordered full diet and porter. One case of incised wound of the wrist, caused by falling on a drawing-knife; the ulnar artery, several of the flexor tendons, and the pisiform bone were divided; the artery had been taken up previous to admission; the hand and arm were flexed; wound brought together with adhesive straps, dressed with cerate and a carved angular splint; wound granulating; ligatures away; still under treatment. One case of lacerated wound of the scalp, caused by a fall; dressed with adhesive strips, since suppurating and poulticed. One case of contusion of the back; ordered perfect rest and frictions. One case of fracture of the neck of the humerus; caused by a fall on the shoulder; dressed with clavicle apparatus.

